# Investigating the Functions of Particles in Packed Aggregate Blend using a Discrete Element Method

**DOI:** 10.3390/ma12040556

**Published:** 2019-02-13

**Authors:** Yinghao Miao, Weixiao Yu, Yue Hou, Liyan Guo, Linbing Wang

**Affiliations:** 1 Beijing Key Laboratory of Traffic Engineering, Beijing University of Technology, Beijing 100124, China; miaoyinghao@ustb.edu.cn (Y.M.); yvweixiao@163.com (W.Y.); gly9825@126.com (L.G.); 2 National Center for Materials Service Safety, University of Science and Technology Beijing, Beijing 100083, China; wangl@vt.edu; 3 Department of Civil and Environmental Engineering, Virginia Tech, Blacksburg, VA 24061, USA

**Keywords:** aggregate mixture, discrete element method, aggregate skeleton structure, mechanical characteristic, interaction forces

## Abstract

In asphalt mixture, aggregates account for up to 90% of the total volume and play an important role in the mechanical characteristics of asphalt mixture. The proportions of fine and coarse aggregates in gradation, as well as the function of aggregate particles, are important factors for the skeleton structure performance of asphalt mixtures. However, the existing asphalt mixture design methods are mostly based on empirical methods, where the non-uniformity and complexity of the composition of asphalt mixtures are not fully studied. In this study, the skeleton structure of aggregate mixture and function of aggregate are studied and analyzed using the Discrete Element Method (DEM). The Particle Flow 3D (PFC3D) DEM program is used to perform the numerical simulation. The average contact number and interaction forces by aggregate particles of different sizes are obtained and studied. The skeleton structure of aggregate mixture and function of aggregate particles are further analyzed from the meso-structural perspective.

## 1. Introduction

At present, the design of asphalt mixtures is mainly based on macro-scale tests, where the macroscopic physical indexes are used as the main criteria for the design of asphalt mixtures, such as gradation of asphalt mixtures, void ratio, crack resistance, fatigue performance, ageing performance, self-healing properties, structure characteristics, moisture and seepage characterization, etc. [1,2,3,4,5,6,7,8,9,10,11,12]. Many of the indicators do not consider the heterogeneity and diversity of the complex internal mineral aggregate mixture structure, and lack systematic research on the aggregate distribution and micro-characteristics of the aggregate mixture skeleton from the perspective of the micro-structure, although there has been some research on the Molecular Dynamics simulation aspect [13,14] for asphalt-based materials. 

Asphalt mixture is a system consisting of a variety of aggregates with different particle sizes according to certain proportions. The geometric characteristics of particles and the proportion of different particle sizes are important factors affecting the mechanical properties of the whole mixtures [15,16,17,18]. The proportion of coarse aggregate to fine aggregate in the mixture determines the skeleton structure and the anti-deformation ability to external loading forces [19,20]. Therefore, in order to further understand the mechanical properties of asphalt mixtures, it is necessary to study the skeleton structure characteristics and aggregate function of aggregate mixtures inside the asphalt mixtures. As a powerful numerical tool, the Discrete Element Method (DEM) can well reflect the inhomogeneity and discontinuity of the internal structure of materials and has been widely used in simulating the mechanical behavior of the granular materials nowadays [6,21,22,23]. In addition, the use of advanced computation-aided mix design can effectively reduce the cost of traditional laboratory tests. 

Many researchers used DEM to study the properties of the mixture constituents (aggregate and asphalt binder). You et al. [24,25] used DEM models to study the asphalt concrete properties. Dondi et al. [26] used DEM simulations to evaluate the effects of grain shape and angularity on aggregate packing. Yu et al. [27] developed a 3D DEM to predict the complex modulus of asphalt mixtures. McDowell et al. [28] used the DEM to simulate the distribution of internal forces. Shen et al. [29] conducted particle packing analysis using the DEM simulation. Chen et al. [30] employed DEM to evaluate aggregate skeleton characteristics. Ma et al. [31] conducted virtual simulation of wheel tests for a rutting deformation prediction. 

In this study, the discrete element method is used to simulate the mechanical behaviors of aggregate mixture. The filling tests on the upper and lower limits and synthetic gradations of SMA-16 and AC-25 are simulated. The reliability of the simulation tests is verified by comparing the simulation tests with the previous published indoor test results [32]. Further studies are considering the packing characteristics of aggregate with different particle size and morphology [33]. The filling status and aggregate function of the mixture are also analyzed. The key particle size is investigated to analyze the skeleton-structure characteristics and the function of coarse and fine aggregates, which provides reference for optimization of aggregate gradation design.

## 2. DEM Simulation of Packed Aggregate Blend

The virtual filling test based on discrete element method (DEM) provides a way to understand the load transfer between particles. PFC3D (Three Dimensional Particle Flow Code) is a three-dimensional DEM software, where all the models built in PFC3D are composed of spherical elements, walls, contacts and bonds. This study uses the 3 Dimensional Particle Flow code (PFC3D) for the simulation of virtual filling test of aggregate blends.

### 2.1. Generation of Virtual Mixture with Given Gradation

For uniformly aggregate-filled mixtures, particles can be considered to be randomly distributed in the mixtures. Therefore, according to the given gradation, a random generation algorithm of spherical elements can be used to generate virtual mixtures. Firstly, the particle placement (filling) space is defined, and the “wall” of a certain shape is defined in PFC3D to ensure that the particle unit is put in a given space range and limit the spillover of the particle unit. Then, within the defined space range, a set of spherical elements with gradation characteristics and given mechanical parameters is randomly generated as a virtual mixture.

When generating virtual mixtures in PFC3D software, first, the average particle size of each size is taken as the diameter, and then the number of particles is calculated according to the total bulk volume of the particle size. Then the spherical unit size adjustment program is loaded to convert the large spherical unit into the spherical unit of random size in the range of the size. The calculated aggregate sphere unit is put into the pre-defined range. After the completion of the deployment, the virtual aggregate volume and the volume of each particle size are calculated. The effectiveness and accuracy of the mix algorithm are verified by comparing the proportion of the volume of each particle size in the pre-determined gradation.

### 2.2. Simulation Plan

For convenience, denote *A* to represent an aggregate blend with given gradation and mass. Denote *A*_s_ as the particles of *A* retained on the sieve with size of *s* but having passed the nearest larger size sieve. For example, *A*_4.75_ represents the particles that have passed the sieve with size of 9.5 mm but are retained on the sieve with size of 4.75 mm. Denote *A*_s-M_ as all the particles of A bigger than s. For example, *A*_4.75-M_ represents all the particles bigger than 4.75 mm in *A*.

In order to verify the reliability of DEM simulation by indoor test, six kinds of gradations used in our previous publication [32] are selected in this paper. They are the reference upper limit, the reference lower limit, and a designed gradation for Stone Matrix Asphalt (SMA) with the Nominal Maximum Particle Size (NMPS) of 16 mm, and dense Asphalt Concrete (AC) with the NMPS of 25 mm, which are denoted as denoted as SMA_U_, SMA_D_, SMA_L_, AC_U_, AC_D_, and AC_L_ [32]. The specific information about the 6 gradations was described in Table 1. In order to quantitatively describe the contribution rate of a certain size particle to the filling volume of the mixture, PCPV (percentage of contribution to the packing volume) is defined in reference [32]. For aggregate mixture with given mass and gradation, it is gradually mixed from large particle size to small particle size. The change of filling volume caused by filling a particle size is recorded as the contribution of the particle size to the filling volume of the mixture. The ratio of the filling volume contribution of the particle size to the total filling volume of the mixture is called the PCPV of the particle size. For more details of PCPV, refer to reference [32]. The packing tests using the Superpave Gyratory Compactor (SGC) to quantify PCPV in reference [32] are simulated by DEM.

Shen et al. [29] and Li [34] simulated aggregate particles with different sizes in DEM simulation, and carried out optimization research on gradation and volume parameters. Chen et al. [30] used DEM to simulate penetration test of asphalt mixture. The model simulated aggregate particles with different sizes of balls. The comparison with indoor penetration test shows that DEM simulation has better reliability. In this simulation, the aggregate particles are also assumed as sphere and simulated using balls of different sizes, and the mass is 3 kg for various gradations, which is consistent with the packing test in laboratory [32]. The space of the particles is defined as a cylinder with a diameter of 150 mm and a height of 500 mm. The wall is used to simulate the filling barrel, the upper and lower pressure indenters. In the simulation test, the normal contact force is determined by Equation (1) and the contact force in the shear direction is calculated by Equation (2) [35].

(1) $$F_{n}^{i}=k_{n}U_{n}^{i}.$$

where $F_{n}^{i}$ is the *i*th normal contact force, *k_n_* is the normal stiffness, $U_{n}^{i}$ is the corresponding normal displacement.

(2) $$\Delta F_{s}^{i}=-k_{s}\Delta U_{s}^{i}$$

where $\Delta F_{s}^{i}$ is the *i*th contact force increment in the shear direction, *k_s_* is the shear stiffness, and $\Delta U_{n}^{i}$ is the corresponding increment of shear displacement.

The normal and shear stiffness depends on the applied contact model. In this paper the build-in linear elastic contact model of PFC3D is employed, where the normal and shear stiffness is determined by the size and elastic modulus (stiffness) of the contacted pieces. The maximum allowable shear contact force is calculated by Equation (3) [35].

(3) $$F_{s}^{\max}=\mu \left|F_{n}^{i}\right|$$

where $F_{s}^{max}$ is the maximum allowable shear contact force, $F_{n}^{i}$ is the *i*th normal contact force, and *μ* is the friction coefficient.

The mechanical parameters of aggregate mainly refer to the existing research results [36,37]. The friction coefficient between pieces is set to 0.2, the elastic modulus of aggregate is 50 GPa. The stiffness of the upper and lower wall panels and the side of cylinder is 500 GPa. The balance size between air voids filling function and skeleton building function is no smaller than 2.36 mm for all the 6 gradations in accordance with reference [32]. With consideration of the efficiency of simulation, *A*_16-M_, *A*_13.2-M_, *A*_9.5-M_, *A*_4.75-M_, *A*_2.36-M_, *A*_1.18-M_, and *A*_0.6-M_ are finally selected as the aggregate blends of the DEM simulation for the 3 SMA gradations. For the 3 AC gradations, *A*_26.5-M_, *A*_19-M_, *A*_16-M_, *A*_13.2-M_, *A*_9.5-M_, *A*_4.75-M_, *A*_2.36-M_, and *A*_1.18-M_ are finally selected. In the virtual filling test, the number of spherical particle units of SMA graded *A*_0.6-M_ of SMA_U_, SMA_D_, and SMA_L_ is 134493, 72296 and 64036, respectively. In the virtual filling test of AC graded *A*_1.18-M_, the number of spherical particle units of AC_U_, AC_D_, and AC_L_ is 53089, 38787 and 31285, respectively. By extracting the filling volume obtained from virtual filling test of aggregate mixtures with different compositions, the PCPV values of particles with different sizes in the mixtures can be calculated.

### 2.3. Simulation Process

**Figure 1:**
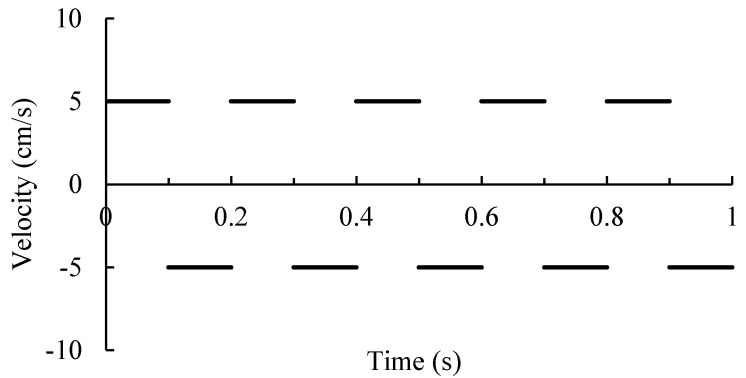
Velocity boundary of the floor for simulation vibration.

**Figure 2:**
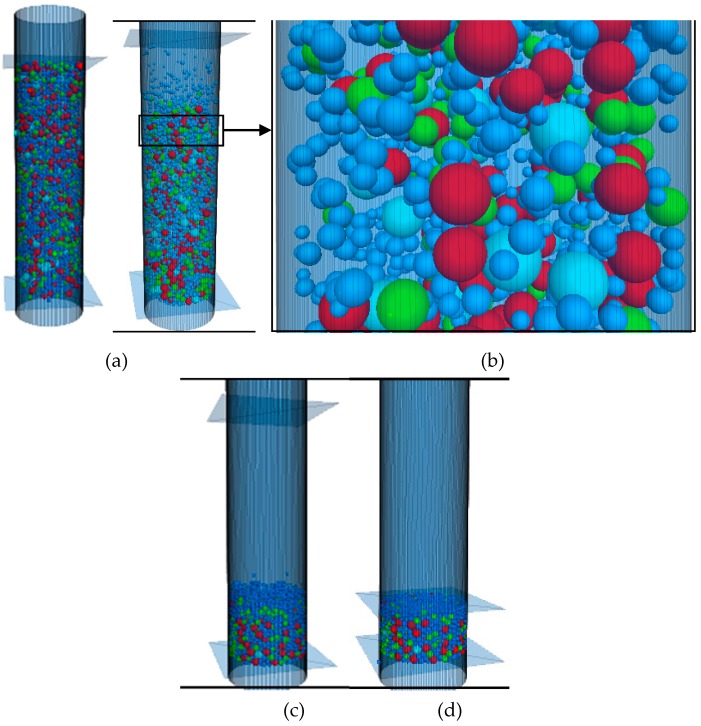
DEM simulation processes: (**a**) Generating particles; (**b**) Falling the particles; (**c**) Before compaction; (**d**) Compacted.

According to the particle composition of each filling test, the corresponding virtual particle mixture is randomly generated. Through the PFC3D subroutine, the aggregate particles generated in the cylinder are subjected to gravity, which make them fall to the bottom of the cylinder. Vibration loads are simulated by applying velocity boundary conditions as shown in Figure 1 to the bottom plate, so as to obtain the aggregate mixture with close contact. Then, the servo compaction subroutine is used to simulate the loading with the upper wall of the cylinder. By specifying the speed of 5 mm/min, it can move downward to simulate the load on the aggregate mixture. At the same time, the contact force of the upper interface of the mixture is monitored. When the vertical force of particles on the roof reaches 2 KN and the given average unbalance rate condition is satisfied, the servo compaction process ends. The height of compacted aggregate mixture is extracted by using PFC3D subroutine. All DEM simulations are computed with automatic time step. After all virtual filling tests are completed, calculate the PCPV for each size particles in the given gradation. The definition and calculation method of PCPV were described in detail in reference [32]. Figure 2 depicts the DEM simulation process.

## 3. Packing Function Analysis

**Figure 3:**
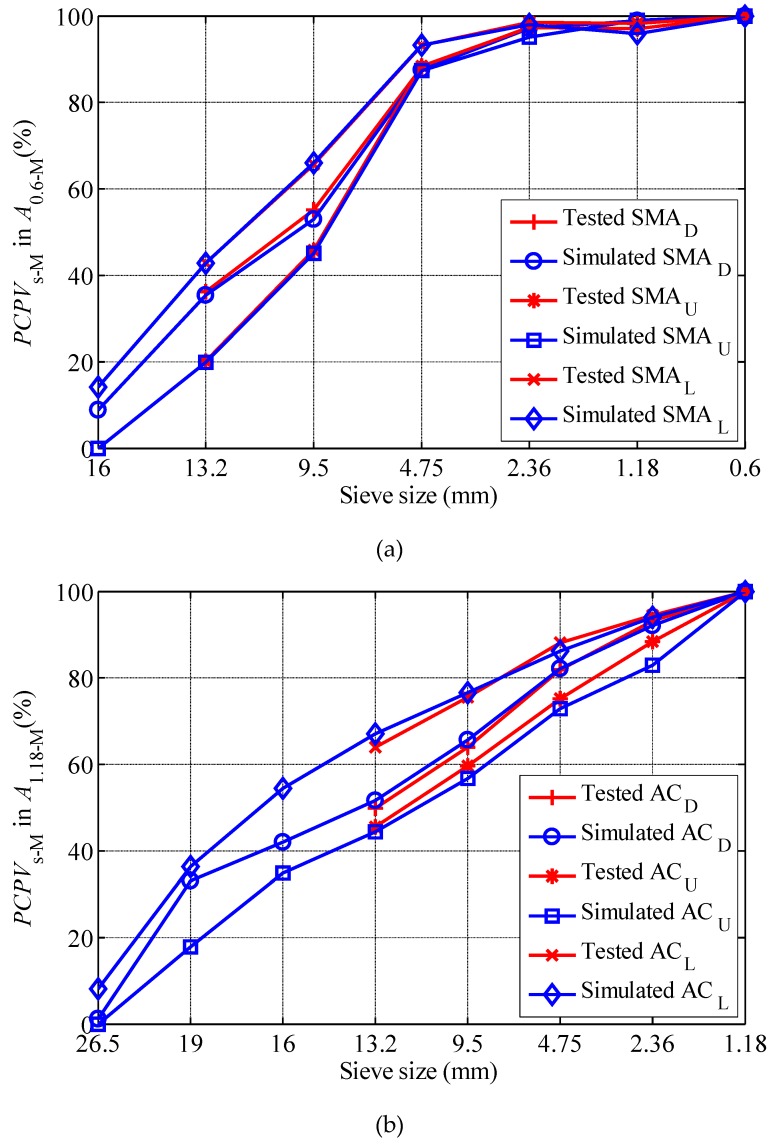
PCPV Comparison between simulation and test: (**a**) SMA; (**b**) AC.

In order to verify the accuracy and reliability of DEM simulation, PCPV of particles with different sizes in *A*_0.6-M_ of SMA and particles with different sizes in *A*_1.18-M_ of AC are calculated by simulation and test, respectively. It should be noted that in the simulated filling test, the filling test started with nominal maximum particle size, adding the next particle size one by one until the final mixture (*A*_0.6-M_ for SMA, *A*_1.18-M_ for AC) is formed. The filling test starts with *A*_13.2-M_, adding the next particle size one by one until the final mixture is formed. Figure 3 depicts the *PCPV*_s-M_ derived from DEM simulation and laboratory test for all gradations. From Figure 3, it can be seen that the PCPV curve obtained by simulation analysis basically coincides with the curve obtained by experiment, indicating that the DEM simulation analysis has good reliability. Based on PCPV test results, the packing function of particles in the 6 gradations was analyzed in detail by reference [32]. The balance size between air voids filling function and skeleton building function were also discussed [32]. In view of the fact that the PCPV correlation results obtained from DEM simulation analysis are basically consistent with those obtained from laboratory tests, the packing function of particles is no longer discussed in detail here, which can be referred to in reference [32].

## 4. Load Transferring Function Analysis

### 4.1. Load Transfer Paths in Aggregate Mixture

**Figure 4:**
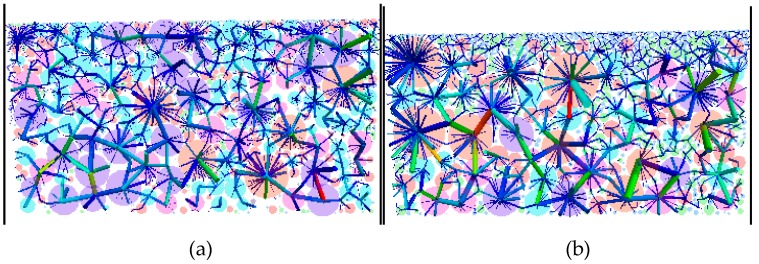
Typical distribution of contact points and contact force: (**a**) SMA_D_; (**b**) AC_D_.

Figure 4 shows the contact point and contact force distribution of SMA-16 synthetized M_0.6_ and AC-25 synthetized M_1.18_. Different colors represent aggregates with different particle sizes. The thicker the force chain is, the large the force is.

Figure 4 shows the internal skeleton structure and the way of load transfer in the mixture. The internal structure of the mixture is actually a network of load transfer by adjacent aggregates. It could be seen that the load transfer in the mixture is priority for large size aggregate or areas, where large size of aggregate distribution is relatively concentrated. Different aggregate sizes have different functions in the mixture and have different contributions in the load transfer. Coarse aggregate usually has more contact numbers, and the contact points are mostly located in the thick force chain. The larger the size of aggregates, the more the number of contact points around aggregates, and the larger the contact force corresponding to the contact points of these particles.

### 4.2. Indicators for Quantifying Load Transferring Features

The aggregate plays a dominant role in the volume and quality of asphalt mixture, and is an important factor affecting the mechanical properties of asphalt mixture. The analysis of load transfer characteristics between particles in aggregate blend is helpful to understand the function of particles and improve the design of aggregate composition of asphalt mixture. In this paper, the coordination number of particles, the scoring coordination number and the contact force of each contact point in the mixture are selected as the parameters to evaluate the load transfer characteristics.

The coordination number is an important micro-structure index reflecting the contact information between particles in the structure of aggregate mixtures. It is defined as the number of contacts between a particle and surrounding particles in the mixture. The number of contacts between a particle and all surrounding particles is defined as the component coordination number. When the aggregate mixture is compacted, the load transfers through the contact points between aggregates. Based on the coordination number, the law of load transfer between the catenary and aggregate particles in the mixture can be preliminarily understood. When the particles are simplified to rigid bodies, the coordination number of particles is one of the main factors that affects the load transfer and deformation in the aggregate mixture. 

The scoring coordination number of *A*_s_ can be calculated by Equation (4), the average coordination number of a single particle $\bar{n_{s}}$ in *A*_s_ can be calculated by Equation (5), and the average contact force of particles in *A*_s_ can be calculated by Equation (6). In the packed aggregate blend, the load bearing contributions of particles in *A*_s_ can be captured by the indirect characterization of the proportion (*P*_s_) of the average contact force of particles in *A*_s_ in the sum of the average contact forces of particles of various sizes in the mixture, and calculated by Equation (7).

(4) $$N_{s}=\sum_{i=1}^{g}n_{s,i}$$

where *g* represents the total particle numbers of *A*_s_, and $n_{s,i}$ is the contact number of the *i*th particle of *A*_s_.

(5) $$\bar{n_{s}}=\frac{N_{s}}{g}$$

(6) $$\bar{F_{s}}=\frac{\sum_{i=1}^{N_{k}}F_{s,i}}{g}$$

where $F_{s,i}$ is the contact force at the *i*th contact point of the particles in *A*_s_.

(7) $$P_{s}=\frac{\bar{F_{s}}}{\sum_{i=1}^{L}\bar{F_{s_{i}}}}$$

where *L* is the total size number in the packed aggregate blend.

### 4.3. Investigation into Load Transferring Function of Particles

**Figure 5:**
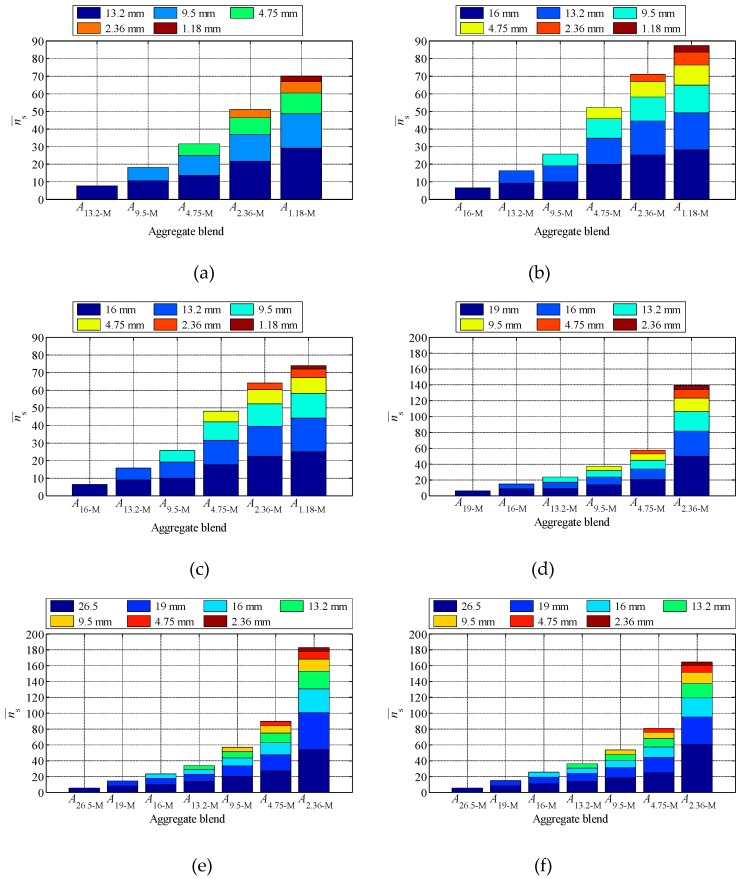
The average coordination number of each size particles in the tested blends: (**a**) SMA_U_; (**b**) SMA_D_; (**c**) SMA_L_; (**d**) AC_U_; (**e**) AC_D_; (**f**) AC_L_.

Figure 5 presents the average coordination number of each size particles in the tested blends. As shown in Figure 5, the addition of particles with smaller size can increase the $\bar{n_{s}}$ of particles with larger size. For example, in SMA_U_, with the addition of the next grade aggregate step by step in blend, $\bar{n_{s}}$ of *A*_13.2_ is 7.75, 10.6, 13.55, 21.78 and 29.09 in *A*_13.2-M_, *A*_9.5-M_, *A*_4.75-M_, *A*_2.36-M_, and *A*_1.18-M_, respectively. In the same mixture, the coordination number and particle size of particles are related, and the coarser particles occupy a larger space and can contact with more particles. Thus, more particles can be contacted and have a larger coordination number.

**Figure 6:**
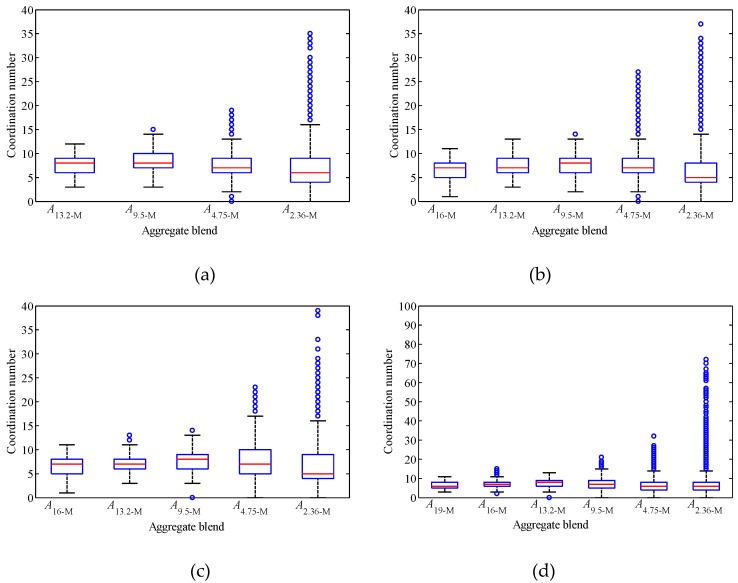

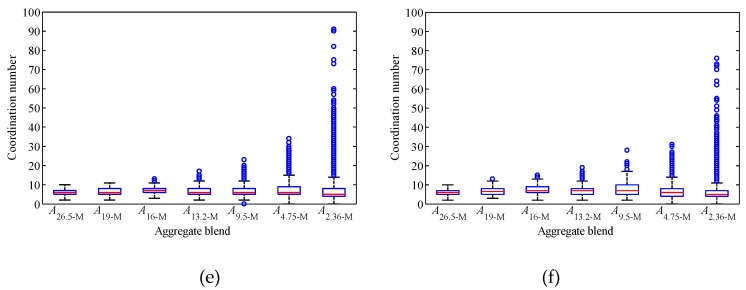
The coordination number distribution of each tested blend: (**a**) SMA_U_; (**b**) SMA_D_; (**c**) SMA_L_; (**d**) AC_U_; (**e**) AC_D_; (**f**) AC_L_.

Figure 6 depicts the coordination number distribution of tested blends by box-and-whisker plot, in which the mark inside the box is the median, the lower and the upper edges of the box are the 1st and 3rd quartiles, respectively, the circles are the outliers. As shown in Figure 6, with the gradual addition of smaller size particles, the distribution range of coordination number of particles in blend increases. This is mainly due to the fact that when the particle size difference increases gradually, large particles can contact more small particles, and the maximum coordination number of particles in the mixture increases with the increase of the particle size difference in the mixture. It can also be found from Figure 6 that although the distribution range of coordination number of particles increases with the gradual addition of smaller size particles, the median of coordination number of particles in mixtures does not always increase with the addition of smaller particles.

The mean and median coordination numbers of all particles in tested blends are listed in Table 2. As shown in Table 2, for the three SMA gradations, the mean and median coordinate numbers of particles in the mixture increase first and then decrease with the addition of smaller size particles. For the three AC gradations, the median coordinate number of particles increases first and then decreases with the addition of smaller size particles. Although the mean coordinate number of particles fluctuates, the overall change is basically the same. Most of the six gradations begin to decrease after 4.75 mm particles are added. When 2.36 mm particles are added, the extent of reduction begins to increase. This change may indicate that the function of the newly added particles has changed, and the load transfer function of the newly added particles may weaken or disappear. In addition, the distribution pattern of coordination number in Figure 6 and the difference between mean and median in Table 2 show that the coordination number is not in a normal distribution, and relevant statistical analysis should pay attention to this problem.

### 4.4. Analysis of Load Bearing Contributions

**Figure 7:**
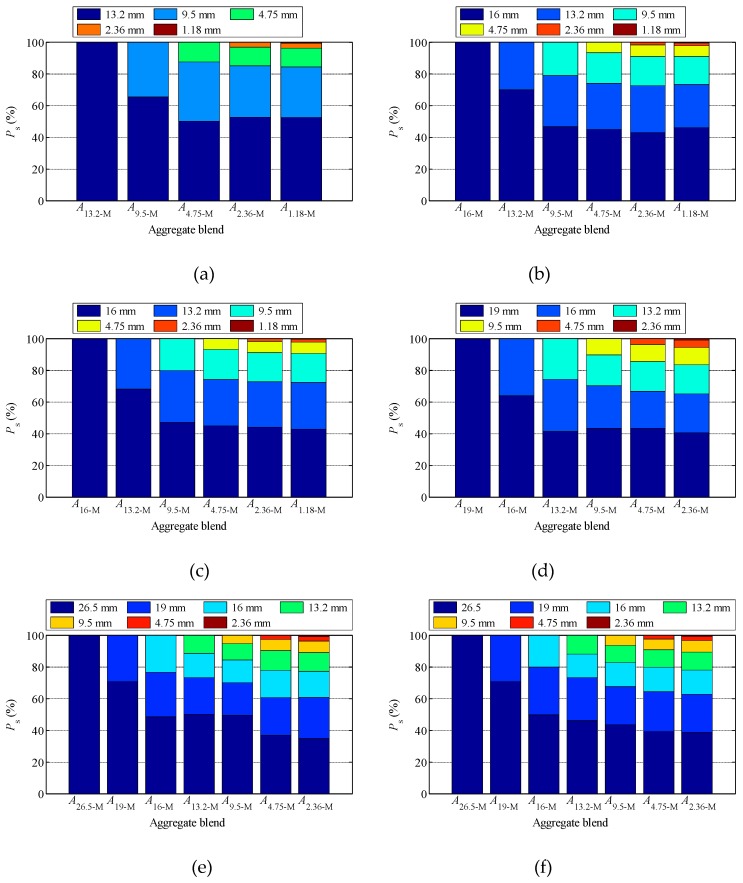
The load bearing contributions of each size particles in the tested blends: (**a**) SMA_U_; (**b**) SMA_D_; (**c**) SMA_L_; (**d**) AC_U_; (**e**) AC_D_; (**f**) AC_L_.

Figure 7 shows the load bearing contributions of each size particles in the tested blends. As shown in Figure 7, with the addition of particles with smaller size, the *P*_s_ of larger particles gradually decreases. For example, in SMA_D_, *P*_s_ of *A*_16_ is 100%, 70.3%, 46.7%, and 44.95% in *A*_16-M_, *A*_13.2-M_, *A*_9.5-M_, and *A*_4.75-M_, respectively, indicating that under external loading conditions, the addition of small particles will help to improve the force conditions of large particles in the aggregate mixture. However, with the addition of particles with smaller sizes, the load sharing effect of newly added particles on larger size particles also decreases gradually.

Table 3 lists *P_s_* values of *A*_9.5_, *A*_4.75_, and *A*_2.36_ in *A*_2.36-M_ blend with each gradation. It can be seen from Table 3 that, for SMA, *P*_s_ of *A*_2.36_ in *A*_2.36-M_ is 3.02%, 1.66%, and 1.66% for SMA_U_, SMA_D_, and SMA_L_ respectively. For 3 AC gradations, *P*_s_ of *A*_2.36_ in *A*_2.36-M_ is no more than 1%. *P*_s_ of *A*_4.75_ in *A*_2.36-M_ is 4.61%, 2.97%, and 2.71% for AC_U_, AC_D_, and AC_L_ respectively. If 5% *P*_s_ is taken as a threshold for function, for SMA gradation, 2.36 mm is the corresponding functional conversion particle size, and 4.75 mm for AC.

### 4.5. Crushing Test

In order to verify the above simulation results of key size particle in the mixture skeleton, the crushing value test (T0316) in Test Methods of Aggregate for Highway Engineering JTG E42-2005 (China) was referred, where the aggregate mixture crushing test of *A*_2.36-M_ blends with gradations of SMA_U_, SMA_D_, and SMA_L_ were conducted. 

**Figure 8:**
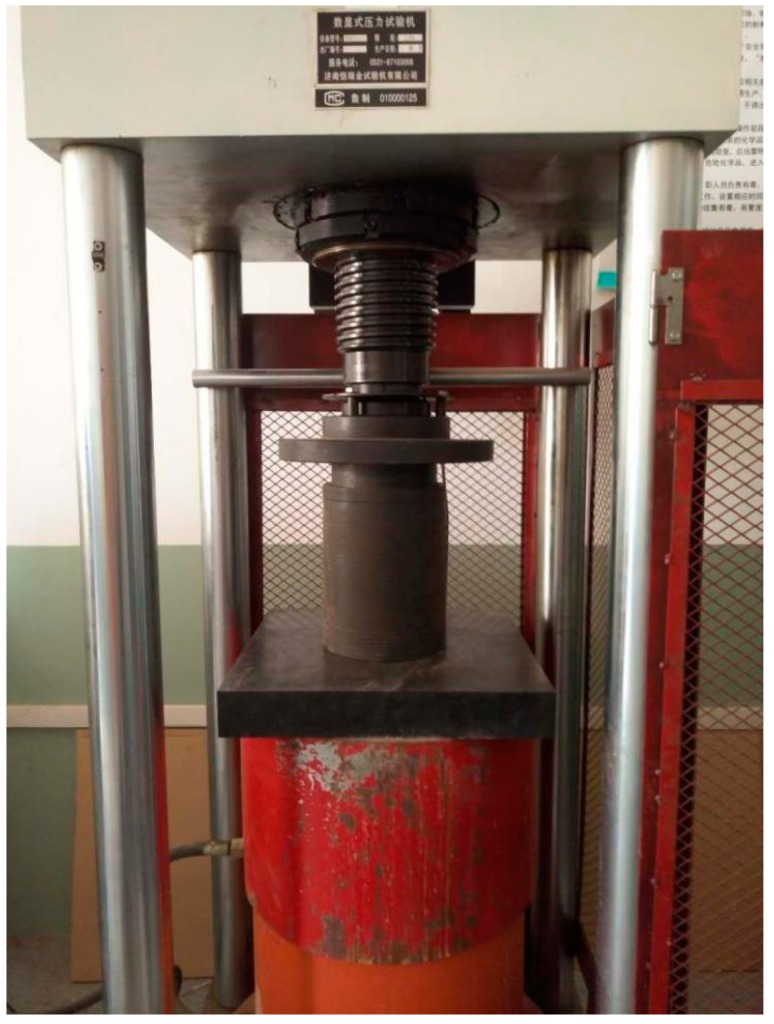
The press machine.

**Figure 9:**
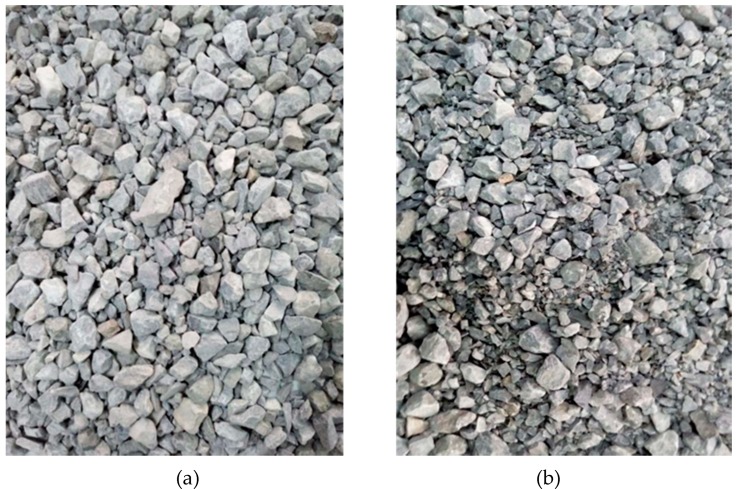
Typical blend before and after crushing test: (**a**) Original; (**b**) Crushed.

**Figure 10:**
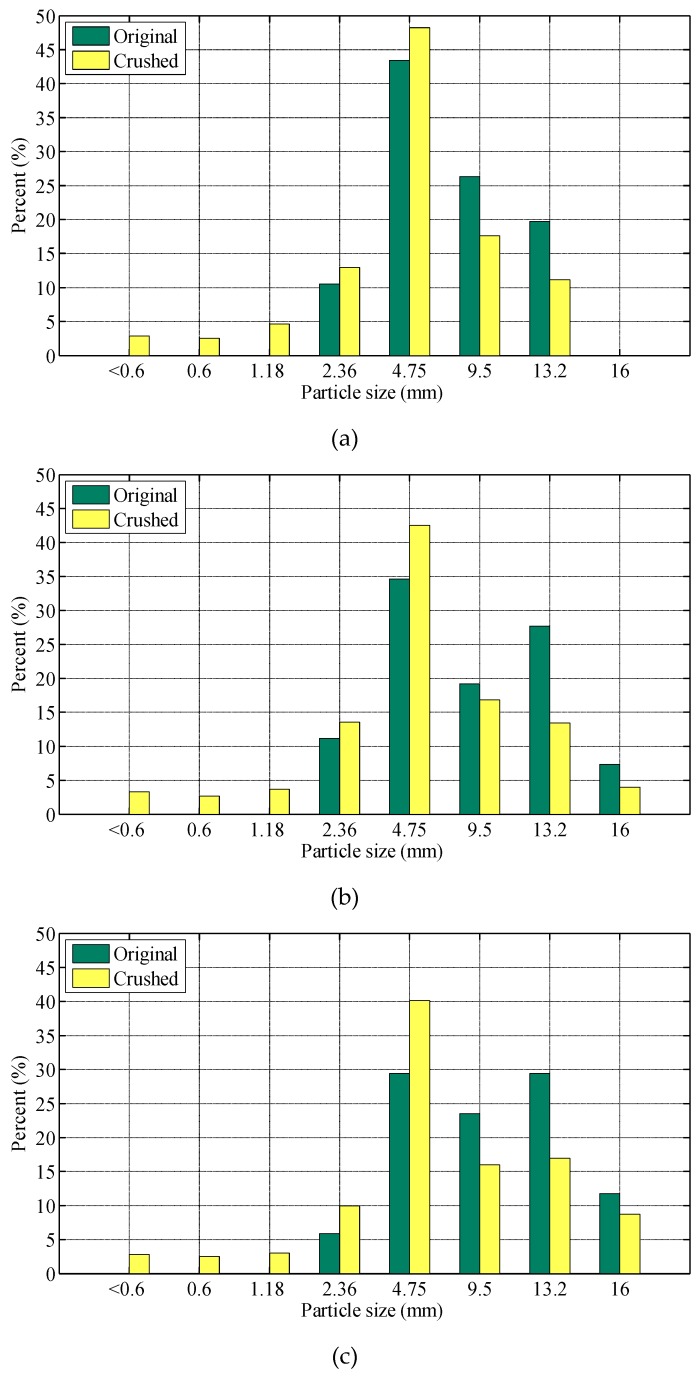
Grain size change of each blend after crushing test: (**a**) SMA_U_; (**b**) SMA_D_; (**c**) SMA_L_.

The crushing test instrument is shown in Figure 8. The total mass of the aggregate mixture was 3 kg. During the test, 3 kg of *A*_2.36-M_ aggregate mixtures were gradually put into the tube by 3 layers, where each layer would be conducted leveling and compaction for 25 times. The crushing test was carried out in 400 KN loading force on the mixture and then crushed and screened. Figure 9 presents a typical blend before and after crushing test. The grain size changes of the blends after crushing test are presented in Figure 10. 

From Figure 10, it can be seen that compared with the particle composition before crushing, the change of the proportion of each particle in the three mixtures is not the same. For SMA_U_, the proportion of 9.5 mm and 13.2 mm particles in the mixture decreases from 26.32% and 19.74%, to 17.63% and 11.13% respectively. For SMA_D_, it decreases from 19.18% and 27.67% to 16.85% and 13.43% respectively. For SMA_L_, it decreases from 23.53% and 29.41% to 15.97% and 16.93% respectively. Among the three graded mixtures, the proportion of 4.75 mm particles increases. Among the three gradations of SMA_U_, SMA_D_ and SMA_L_, the proportion of 4.75 mm particles increases by 4.81%, 7.92% and 10.75% respectively. The proportion of particles less than 2.36 mm in the three gradations also increases significantly.

The pressure crushing process of mixtures is the result of load transfer between particles in mixtures. The crushing of particles can indirectly reflect the load bearing contributions of particles. The increase of the proportion of particles below 4.75 mm after crushing test indicates that the load shared by these particles in crushing test is too small to cause particle crushing. The increase of the proportion of particles above 9.5 mm after crushing test indicates that these particles share a larger load in the test, resulting in the crushing of particles. As shown in Table 3, for SMA_U_, SMA_D_, and SMA_L_, the *P_s_* values of A_2.36_ in A_2.36-M_ are 3.02%, 1.66% and 1.66% respectively, indicating that A_2.36_ only shares a very small part of the load. For S SMA_U_, SMA_D_, and SMA_L_, the *P_s_* values of A_4.75_ in A_2.36-M_ are 11.84%, 7.23%, and 7.21%, respectively, indicating that A_4.75_ has a certain load-bearing function, but its load-sharing is still relatively small compared with 9.5 mm and above particles, which coincides with the increase of the proportion of particles under 4.75 mm in the test. Among the three SMA gradations, the proportion of load shared by particles under 4.75 mm in SMA_U_ is the largest, which also means that the proportion of load shared by A_9.5-M_ in SMA_U_ is the smallest. In the corresponding crushing experiments, the reduction rate of particles above 9.5 mm should be lower than that of the other two gradations, which is exactly consistent with the crushing experiment results. From the above-mentioned crushing law of mixtures, it can be further explained that in the crushing process of mixtures, large particles are the main medium of load transfer in the mixture and the main particle size of the skeleton.

## 5. Conclusions

In this study, the skeleton structure of aggregate mixture and the function of aggregate are studied and analyzed using the Discrete Element Method (DEM). The Particle Flow 3D (PFC3D) DEM program is used to perform the numerical simulation. The following conclusions can be obtained:

The comparisons between PFC3D simulation results and indoor filling test/crushing test show that it is feasible to study the filling structure of mineral mixture using DEM simulation. DEM simulation can not only investigate the filling function of particles in mineral mixture, but also analyzes the load transfer function of particles in mineral mixture based on the coordination number and contact force information. It is an effective approach to understand the function of particles in mineral mixture.

With the addition of small and medium size particles in the mineral mixture, the proportion of sharing load by large size particles gradually decreases, which is helpful to improve the stress condition of larger particles in the mixture. However, as the particle size becomes smaller, the load-sharing effect of new particles on larger particles decreases rapidly. The load transfer function of particles can be quantitatively evaluated by the proportion of load shared by particles in mineral mixture.

The skeleton building and air voids filling functions are evaluated by the proportion of particles in the mineral mixture. For SMA-16 gradation, 2.36 mm is the main function conversion particle size. For AC-25 gradation, the main function conversion particle size is 4.75 mm.

There are significant differences in load transfer function between particles with different sizes in SMA and AC graded aggregate mixtures. Quantitative analysis of load transfer function of particles provides a powerful tool for further understanding the influences of aggregate composition on asphalt mixtures performance, which has an application potential in design optimization of aggregate mixtures and development of new gradation.

## Figures and Tables

**Table 1:** The selected gradations [32].

Seive Size (mm)	Percent Passing (%)					
	SMA_U_	SMA_D_	SMA_L_	AC_U_	AC_D_	AC_L_
31.5	-	-	-	100	100	100
26.5	100	100	100	100	99.3	95
19	100	99.7	100	90.0	79.1	75
16	100	94.0	90	80	72.2	62
13.2	85	71.5	65	73	65.3	53
9.5	65	55.9	45	63	52.5	43
4.75	32	27.8	20	52	38.9	32
2.36	24	18.7	15	42	28.8	25
1.18	22	16.2	14	32	20.6	18
0.6	18	14.2	12	25	14.5	13
0.3	15	12.8	10	18	10.1	8
0.15	14	12.0	9	13	7.6	5
0.075	12	11.2	8	7	6.5	3

**Table 2:** The mean and median coordination numbers of all particles in tested blends.

Blends		*A*_26.5-M_	*A*_19-M_	*A*_16-M_	*A*_13.2-M_	*A*_9.5-M_	*A*_4.75-M_	*A*_2.36-M_
SMA_U_	Mean	/	/	/	7.75	8.38	7.70	7.04
	Median	/	/	/	8	8	7	6
SMA_D_	Mean	/	/	6.54	7.34	7.85	8.00	6.27
	Median	/	/	7	7	8	7	5
SMA_L_	Mean	/	/	6.54	7.13	7.79	7.85	6.82
	Median	/	/	7	7	8	7	5
AC_U_	Mean	/	6.23	7.01	7.81	7.34	6.43	6.81
	Median	/	6	7	8	7	6	6
AC_D_	Mean	5.59	6.57	7.06	6.61	7.33	7.57	6.62
	Median	6	6	7	6	6	6	5
AC_L_	Mean	5.59	6.71	7.42	7.23	7.77	7.05	6.34
	Median	6	6.5	7	7	7	6	5

**Table 3:** The *P*_s_ values of *A*_9.5_, *A*_4.75_, and *A*_2.36_ in *A*_2.36-M_ blend with each gradation.

Gradation	SMA_U_	SMA_D_	SMA_L_	AC_U_	AC_D_	AC_L_
*A*_9.5_	32.36%	18.47%	18.25%	10.91	7.29%	7.25%
*A*_4.75_	11.84%	7.23%	7.21%	4.61%	2.97%	2.71%
*A*_2.36_	3.02%	1.66%	1.66%	0.91%	0.59%	0.55%

## List of Defined Acronyms

DEM: discrete element method
PFC3D: the particle flow 3D
SGC: Superpave gyratory compactor
*A*: an aggregate blend with given gradation and mass
*A*_s_: the particles of *A* retained on the sieve with size of *s* but having passed the nearest larger size sieve
*A*_s-M_: all the particles of *A* bigger than the size of s
NMPS: the nominal maximum particle size
SMA: stone matrix asphalt
AC: asphalt concrete
SMA_U_: the reference upper limit of SMA gradation
SMA_D_: the designed SMA gradation
SMA_L_: the reference lower limit of SMA gradation
AC_U_: the reference upper limit of AC gradation
AC_D_: the designed AC gradation
AC_L_: the reference lower limit of AC gradation
SMA-16: SMA with the NMPS of 16 mm
AC-25: AC with the NMPS of 25 mm
PCPV: percentage of contribution to the packing volume
*PCPV*_s-M_: the total PCPV of particles bigger than the size of s
$n_{s,i}$: the contact number of the *i*th particle of *A*_s_.
*N_s_*: The scoring coordination number of *A*_s_
$\bar{n_{s}}$: the average coordination number of a single particle in *A*_s_
g: the total particle numbers of *A*_s_
$F_{s,i}$: the contact force at the *i*th contact point of the particles in *A*_s_
$\bar{F_{s}}$: the average contact force of particles in *A*_s_
*P_s_*: the proportion of the average contact force of particles in *A*_s_ in the sum of the average contact forces of particles of all sizes in the mixture
*L*: the total size number in the packed aggregate blend

## References

[1] Fuller W.B., Taylor F.W., Thompson S.E. (1906). A Treatise on Concrete, Plain and Reinforced: Materials, Construction, and Design of Concrete and Reinforced Concrete.

[2] Vavrik W.R., Pine W.J., Huber G., Carpenter S.H. (2001). The Bailey method of gradation evaluation: The influence of aggregate gradation and packing characteristics on voids in the mineral aggregate. J. Assoc. Asph. Paving Technol..

[3] Zhang X., Wang S., Wu K., Wang D. (2001). The CAVF method of asphalt mixture composition design. Highway.

[4] Kim S., Guarin A., Roque R., Birgisson B. (2008). Laboratory evaluation for rutting performance based on the DASR porosity of asphalt mixture. Road Mater. Pavement Des..

[5] Chen H., Xu Q., Chen S., Zhang Z. (2009). Evaluation and design of fiber-reinforced asphalt mixtures. Mater. Des..

[6] Liu Y., You Z., Li L., Wang W. (2013). Review on advances in modeling and simulation of stone-based paving materials. Constr. Build. Mater..

[7] Zaumanis M., Poulikakos L.D., Partl M.N. (2018). Performance-based design of asphalt mixtures and review of key parameters. Mater. Des..

[8] Luo X., Gu F., Lytton R.L. (2015). Prediction of Field Aging Gradient in Asphalt Pavements. Transp. Res. Rec..

[9] Xu H., Xing C., Zhang H., Li H., Tan Y. (2019). Moisture seepage in asphalt mixture using X-ray imaging technology. Int. J. Heat Mass Transf..

[10] Xu H., Guo W., Tan Y. (2015). Internal structure evolution of asphalt mixtures during freeze-thaw cycles. Mater. Des..

[11] Luo X., Lytton R.L. (2016). Characterization of Healing of Asphalt Mixtures Using Creep and Step-Loading Recovery Test. J. Test. Eval..

[12] Xu H., Chen F., Yao X., Tan Y. (2018). Micro-scale moisture distribution and hydrologically active pores in partially saturated asphalt mixtures by X-ray computed tomography. Constr. Build. Mater..

[13] Yao H., Dai Q., You Z. (2015). Chemo-physical analysis and molecular dynamics (MD) simulation of moisture susceptibility of nano hydrated lime modified asphalt mixtures. Constr. Build. Mater..

[14] Yao H., Dai Q., You Z. (2016). Molecular dynamics simulation of physicochemical properties of the asphalt model. Fuel.

[15] Masad E., Little D., Sukhwani R. (2004). Sensitivity of HMA Performance to Aggregate Shape Measured Using Conventional and Image Analysis Methods. Road Mater. Pavement Des..

[16] Sengoz B., Onsori A., Topal A. (2014). Effect of aggregate shape on the surface properties of flexible pavement. KSCE J. Civ. Eng..

[17] Bessa I.S., Branco V.T.C., Soares J.B., Neto J.A.N. (2015). Aggregate shape properties and their influence on the behavior of hot-mix asphalt. J. Mater. Civ. Eng..

[18] Gao J., Wang H., Bu Y., You Z., Mohd R.M., Irfan M. (2018). Effects of coarse aggregate angularity on the microstructure of asphalt mixture. Constr. Build. Mater..

[19] Chen J., Huang X. (2012). Evaluation of aggregate skeleton structure using the discrete element method. J. Southeast Univ..

[20] Chen J.S., Zeng L., Yin J. (2013). Discrete element method (DEM) analyses of hot-mix asphalt (HMA) mixtures compaction and internal structure. Adv. Mater. Res..

[21] Hou S., Zhang D., Huang X., Zhao Y. (2015). Investigation of micro-mechanical response of asphalt mixtures by a three-dimensional discrete element model. J. Wuhan Univ. Technol..

[22] Yang X., You Z., Jin C., Wang H. (2016). Aggregate representation for mesostructure of stone based materials using a sphere growth model based on realistic aggregate shapes. Mater. Struct..

[23] Huang K., Xu T., Li G., Jiang R. (2016). The feasibility of DEM to analyze the temperature field of asphalt mixture. Constr. Build. Mater..

[24] You Z., Buttlar W.G. (2004). Discrete element modeling to predict the modulus of asphalt concrete mixtures. J. Mater. Civ. Eng..

[25] You Z., Buttlar W.G. (2005). Application of discrete element modeling techniques to predict the complex modulus of asphalt—Aggregate hollow cylinders subjected to internal pressure. Transp. Res. Rec..

[26] Dondi G., Simone A., Vignali V., Manganelli G. (2012). Discrete element modelling of influences of grain shape and angularity on performance of granular mixes for asphalts. Procedia—Soc. Behav. Sci..

[27] Yu H., Shen S. (2013). A micromechanical based three-dimensional DEM approach to characterize the complex modulus of asphalt mixtures. Constr. Build. Mater..

[28] McDowell G.R., Bolton M.D. (1998). On the micromechanics of crushable aggregates. Geotechnique.

[29] Shen S., Yu H. (2011). Characterize packing of aggregate particles for paving materials: Particle size impact. Constr. Build. Mater..

[30] Chen J., Li H., Wang L.B. (2015). Micromechanical characteristics of aggregate particles in asphalt mixtures. Constr. Build. Mater..

[31] Ma T., Zhang D., Zhang Y., Hong J. (2016). Micromechanical response of aggregate skeleton within asphalt mixture based on virtual simulation of wheel tracking test. Constr. Build. Mater..

[32] Miao Y., Wang S., Guo L., Li J. (2018). A method for quantifying the packing function of particles in packed aggregate blend. Constr. Build. Mater..

[33] Miao Y., Liu X., Hou Y., Li J., Wu J., Wang L. (2019). Packing characteristics of aggregate with consideration of particle size and morphology. Appl. Sci..

[34] Li Y. (2014). Digital Mix Design for Performance Optimization of Asphalt Mixture. Ph.D. Thesis.

[35] Yu H. (2012). Design and Characterization of Asphalt Mixtures Based on Particle Packing and Mechanical Modeling. Ph.D. Thesis.

[36] You Z., Adhikari S., Dai Q. (2008). Three-Dimensional Discrete Element Models for Asphalt Mixtures. J. Eng. Mech..

[37] Liu Y., Dai Q., You Z. (2009). Viscoelastic Model for Discrete Element Simulation of Asphalt Mixtures. J. Eng. Mech..

